# Clinical parameters as predictors for sperm retrieval success in azoospermia: experience from Indonesia

**DOI:** 10.12688/f1000research.141969.1

**Published:** 2023-12-06

**Authors:** Rinaldo Indra Rachman, Ghifari Nurullah, Widi Atmoko, Nur Rasyid, Sung Yong Cho, Ponco Birowo

**Affiliations:** 1 Department of Urology, Faculty of Medicine, Cipto Mangunkusumo Hospital, Universitas Indonesia, Depok, West Java, 10430, Indonesia; 2 Department of Urology, College of Medicine, Seoul National University Hospital, Seoul National University, Gwanak-gu, Seoul, South Korea

**Keywords:** Sperm Retrieval, Azoospermia, FSH, LH, Testosterone, Varicocele, Longest Testicular Axis

## Abstract

**Background:**

Azoospermia is the most severe type of male infertility. This study aimed to identify useful clinical parameters to predict sperm retrieval success. This could assist clinicians in accurately diagnosing and treating patients based on the individual clinical parameters of patients.

**Methods:**

A retrospective cohort study was performed involving 517 patients with azoospermia who underwent sperm retrieval in Jakarta, Indonesia, between January 2010 and April 2023. Clinical evaluation and scrotal ultrasound, serum follicle stimulating hormone (FSH), luteinizing hormone (LH), and testosterone levels were evaluated before surgery. Multivariate analyses were conducted to determine clinical parameters that could predict overall sperm retrieval success. Further subgroup analysis was performed to determine the factors that the diagnosis of non-obstructive azoospermia (NOA) diagnosis and sperm retrieval success among patients with NOA.

**Results:**

A total of 2,987 infertile men attended our clinic. Men with azoospermia (n=517) who met the inclusion criteria and did not fulfil any exclusion criteria were included in the study. The overall sperm retrieval success was 47.58%. Logistic regression revealed that FSH 7.76 mIU/mL (sensitivity: 60.1%, specificity: 63.3%, p<0.001); longest testicular axis length 3.89 cm (sensitivity: 33.6%, specificity: 41.6%); and varicocele (p<0.001) were independent factors for overall sperm retrieval. The FSH cutoff of 7.45 mIU/mL (sensitivity: 31.3%, specificity: 37.7%, p<0,001); longest testicular axis length 3.85 cm (sensitivity: 76.7%, specificity: 65.4%, p<0.001); and varicocele (p<0.001) were independent factors for NOA diagnosis. Varicocele was the only clinical parameter that significantly predicted the success of sperm retrieval in patients with NOA.

**Conclusions:**

FSH, LH, longest testicular axis, and varicocele are among the clinical parameters that are useful for predicting overall sperm retrieval success and NOA diagnosis. However, varicocele is the only clinical parameter that significantly predicts sperm retrieval success in patients with NOA. High-quality studies are required to assess the other predictors of sperm retrieval success.

## Introduction

Infertility is defined as the inability of a sexually active couple to achieve a spontaneous pregnancy within one year without the use of contraception. ^1^ Furthermore, the incidence of infertility is increasing annually. A meta-analysis of infertility data from 1990 to 2017 showed an increase in the prevalence of infertility by age in 195 countries: 0.370% per year for women and 0.291% per year for men. In addition, age-adjusted infertility (DALYs) also increased by 0.396%. per year for women and 0.293% for men. This was observed in all countries. ^2^

Infertility in couples can be caused by the man, woman or both. Of all infertility cases, men play a role in 40–50% of cases. In the Middle East, men exhibited the highest infertility prevalence (60–70%), whereas the lowest prevalence was observed in Asia (37%). ^3^ Azoospermia is the lack of sperm in the ejaculate. Among all male infertility cases, azoospermia is the most complex diagnosis. A comprehensive clinical examination including history taking, physical examination, hormonal evaluation, scrotal ultrasonography, and Y-chromosome microdeletion testing are among the clinical parameters that are important for azoospermia diagnosis. ^1^^,^^4^^,^^5^

The European Association of Urology guidelines recommend two sperm analyses to diagnose azoospermia. ^1^ Subsequently, crucial decisions are required to guide couples along the path to parenting. The couple should be informed that sperm retrieval and assisted reproduction, including traditional adoption, embryo adoption, and donor or partner sperm use are options that may be explored. ^6^^,^^7^

The success rate of sperm retrieval varies among studies. The success rate of conventional testicular sperm extraction (TESE) is 16.7–49%, whereas the success rate of microdissection testicular sperm extraction (mTESE), the gold standard for sperm retrieval in non-obstructive azoospermia (NOA) is 41–63%. ^6^ The success rates of sperm retrieval have been predicted in many studies. ^8^^–^^10^ High levels of follicle stimulating hormone (FSH) have been linked to unsuccessful sperm retrieval, according to studies by Ghalayini *et al.*, and Colpi *et al.*, Further, Colpi *et al.*, reported that there was no real relationship between testicular volume and sperm retrieval, whereas, Ghalayini *et al.*, demonstrated an association between testicular volume and the successful sperm retrieval. ^8^^,^^9^ According to a large cohort study by Ramasamy *et al.,* ^10^ FSH levels are not related to the success of sperm retrieval when sperm are collected using mTESE. Another study reported that testicular biopsy results are the most reliable predictor of sperm retrieval success. However, testicular biopsies are not always available before sperm retrieval. Patients with the most severe testicular histology have a sperm retrieval rate of 5–24%, whereas patients with the least severe form, hypospermatogenesis, achieve a sperm retrieval rate of 80–98%. ^11^ However, to our knowledge, no study has examined the clinical parameters predicting sperm retrieval success in Indonesia.

## Methods

This retrospective cohort study aimed to determine the clinical parameters that predict the success of sperm retrieval. All patients who underwent sperm retrieval procedures performed by the author in Jakarta, Indonesia, between January 2010 and April 2023 were included in this study. Before sperm retrieval, the clinical parameters of patients were assessed. Data were collected retrospectively from medical records containing information that is routinely collected by the authors. Data collection began on 1 May 2023 in two hospitals, including Cipto Mangunkusumo Hospital and Bunda General Hospital Jakarta. The study protocol was approved by the Ethics Committee of the Faculty of Medicine, Universitas Indonesia (Approval no: 23-02-0168 6 March 2023), which is the only ethics committee covering the province of Jakarta and both Cipto Mangunkusumo Hospital and Bunda General Hospital Jakarta hospitals are teaching hospitals of Faculty of Medicine Universitas Indonesia. Data were collected from academic teaching hospitals. Informed consent was provided in the Initial patient registration form, stating that every patient data collected at the hospital are eligible to be published in an academic publication anonymously. Therefore, consent was obtained from each subject and this study was conducted in accordance with the Declaration of Helsinki.

The clinical parameters of the 517 patients were collected and retrospectively analyzed. Clinical evaluation, scrotal ultrasound to evaluate varicocele presence and the longest testicular axis, serum follicle stimulating hormone (FSH), luteinizing hormone (LH), and testosterone levels were considered before surgery. Varicocele was diagnosed based on Sarteschi Classification system for Varicocele. The longest testicular axis was measured in centimeters (cm). All surgeries were performed by the author (PB).

The inclusion criteria for this study were male infertility presenting with azoospermia, meeting the American Society of Anesthesiologists (ASA) grade I-III criteria, and the availability of complete data. The exclusion criteria were severe cardiac or pulmonary insufficiency, severe coagulation disorders, no history of sperm retrieval procedure, incomplete data, and refusal to provide consent for surgery.

### Routine sperm retrieval procedure

Every patient was counselled at the urology clinic about the risks and benefits of sperm retrieval. The patients who decided to undergo sperm retrieval were informed about the following procedures: percutaneous epididymal sperm aspiration (PESA), followed by testicular sperm extraction (TESE) only if sperm were not present after PESA.

The patient was placed in a supine position and fentanyl 1–2 mcg/kg, propofol 1 mg/kg, rocuronium 0.1 mg/kg were administered for general anesthesia. Asepsis and antisepsis of the operative field and surrounding areas were performed using povidone-iodine. The first step involved localization of the epididymis with the operator’s non-dominant hand using a three-finger maneuver, followed by aspiration of the epididymis using a 10 cc syringe. The aspirate was then analyzed under a microscope to identify sperm. If sperm were found, the surgery was complete. However, if sperm were not found, TESE was performed.

The first step of TESE involves incision of the scrotal median raphe, followed by dissection of the tunica dartos and vaginalis of the testis. Bleeding was controlled using electrocoagulation. When the testis was fully exposed, a longitudinal incision (1–2 cm) was created. A small portion of testicular tissue was extracted for microscopic analysis. The testicular incision was sutured using a monofilament nonabsorbable 5.0 cutting needle, while the tunica dartos and vaginalis of the testis were sutured using a multifilament absorbable 4.0 tapered needle. The scrotum was continuously sutured. Intravenous (IV) dexketoprofen (50 mg) and IV granisetron (1 mg) were administered postoperatively.

### Statistical analysis

IBM SPSS Statistics (RRID:SCR_016479) 25.0 Software (IBM Corp., Armonk, NY, USA) was used for the statistical analyses. Categorical data are presented using n (%). Continuous data are presented as the mean ± SD if the distribution is normal and median (min–max) for non-normal distribution. The Kolmogorov Smirnoff single sample test was used to determine normal distribution of continuous variables. Chi-square tests were performed for bivariate analyses. However, if the chi-square criteria were not met, Fisher’s exact test was performed. Statistical significance was set at p<0.05. Further, multivariate analysis using linear regression was performed for eligible variables to analyze the statistical significance of the indicators.

We performed two subgroup analyses. The first subgroup analysis was used to confirm obstructive azoospermia (OA) or NOA diagnosis and the associated predictive factors. OA was confirmed by a successful PESA procedure; otherwise, NOA was diagnosed. The second subgroup analysis aimed to determine the predictive factors for successful sperm retrieval in patients with NOA. Similar statistical analyses were performed for both subgroups.

Receiver operating curve (ROC) analysis was done for clinical parameters significant in each subgroup. Area under the curve (AUC) is a measure of predictive power. A p value of <0.05 was considered statistically significant.

## Results

A total of 517 men with azoospermia (OA: 164, NOA: 353) were included in this study. Patient characteristics are presented in Table 1. ^22^ The mean age of the patients in the successful retrieval group was 37 years old. Overall sperm retrieval success was 47.58% and sperm retrieval success among patients with NOA was 30.62%. The Kolmogorov–Smirnov normality test showed that none of the numeric variables were normally distributed. Bivariate analysis revealed significant differences in varicocele, serum FSH, serum LH, and the longest testicular axis affecting sperm retrieval success. Age and duration of marriage were similar between the groups. Diabetes and history of undescended testes (UDT) were not associated with sperm retrieval success.

**Table 1.** Bivariate analysis for potential clinical parameters to predict sperm retrieval success.

Clinical parameters		Successful sperm retrieval (n=245)	Unsuccessful sperm retrieval (n=271)	p-value
**Age (years)**		37 (22–67)	36 (25–65)	0.555 ^a^
**Marriage duration (years)**		5 (1–20)	5 (1–25)	0.896 ^a^
**Diabetes**	**Yes**	11 (57.89)	8 (42.11)	0.484 ^b^
	**No**	234 (47.08)	263	
**UDT**	**Yes**	7 (50)	7 (50)	1.000 ^b^
	**No**	238 (47.41)	264 (52.92)	
**Varicocele**	**Yes**	75 (26.50)	205 (73.50)	<0.001 ^b^
	**No**	170 (72.03)	66 (27.97)	
**FSH (mg/dL)**		6.59 (0.66–22.40)	11.8 (0.11–69.10)	<0.001 ^a^
**LH (mg/dL)**		4.50 (0.10–12.85)	5.01 (0.07–43.15)	<0.001 ^a^
**Testosterone (ng/mL)**		3.93 (0.02 – 8.20)	3.47 (0.03–7.91)	0.128 ^a^
**Longest testicular axis (cm)**		5.00 (1.31–8.89)	3.47 (1.21–8.61)	<0.001 ^a^

Abbreviations: UDT, undescended testicle; FSH, follicle-stimulating hormone; LH, luteinizing hormone.

^a^ Mann–Whitney test.

^b^ Chi-square test.

Further, logistic regression analysis was performed. FSH level, varicocele, and longest testicular axis were independent predictors of sperm retrieval success ( Table 2). The R ^2^ score for this linear regression was 0.410 indicating that this analysis comprises 41.0% of all the factors affecting sperm retrieval success.

**Table 2.** Linear regression analysis for potential clinical parameters to predict sperm retrieval success.

Clinical parameters	Coefficient	OR	95% CI	p-value
**FSH**	-0.091	0.913	0.886–0.940	<0.001
**LH**	0.033	1.034	0.975–1.096	0.261
**Longest testicular axis**	0.181	1.198	1.054–1.362	0.006
**Varicocele**	-1.964	0.140	0.091–0.217	<0.001

Abbreviations: FSH, Follicle stimulating hormone; LH, luteinizing hormone; OR, odds ratio; CI, confidence interval.

R ^2^=0.410.

**Figure 1.**
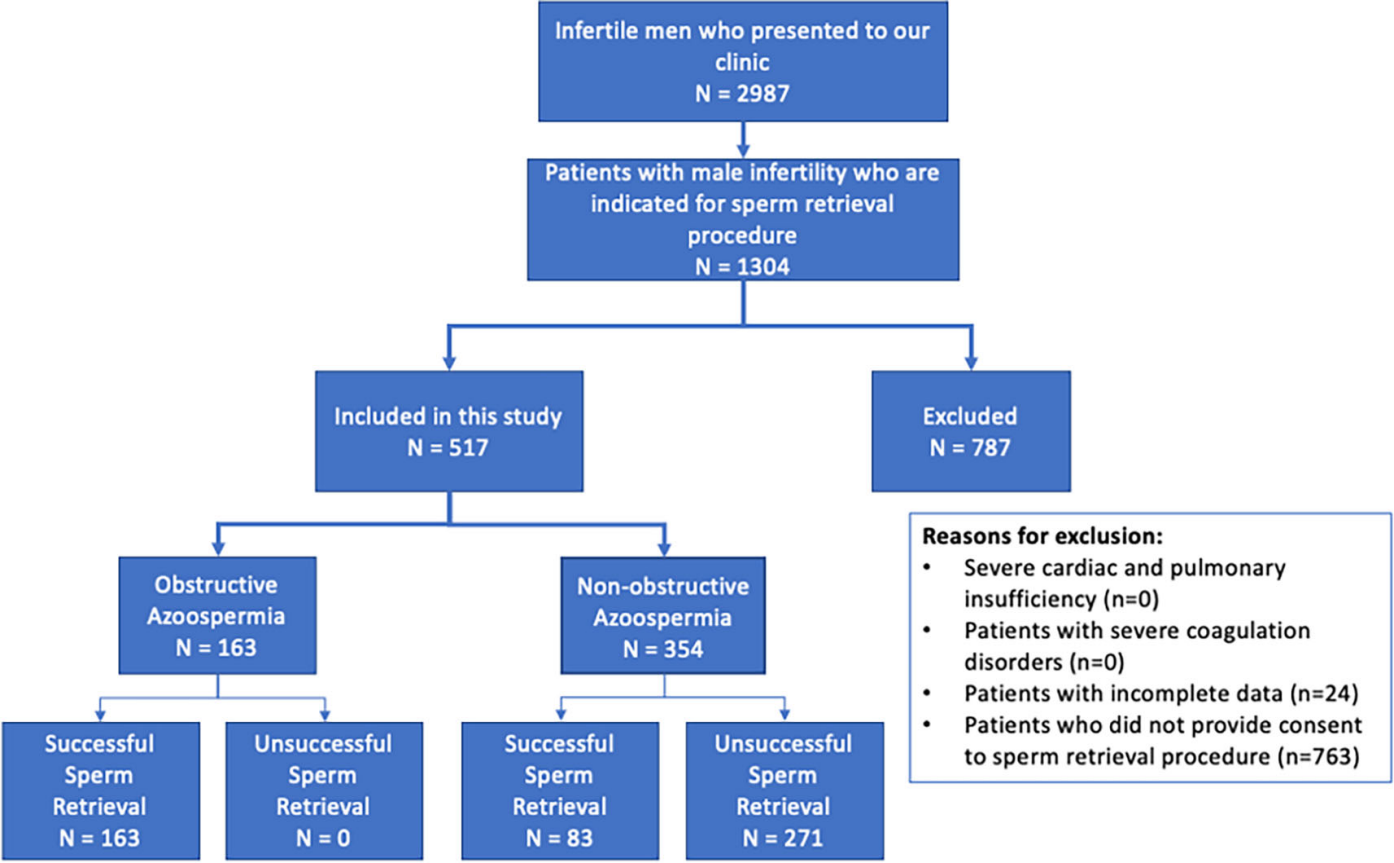
Participant recruitment flowchart.

**Figure 2.**
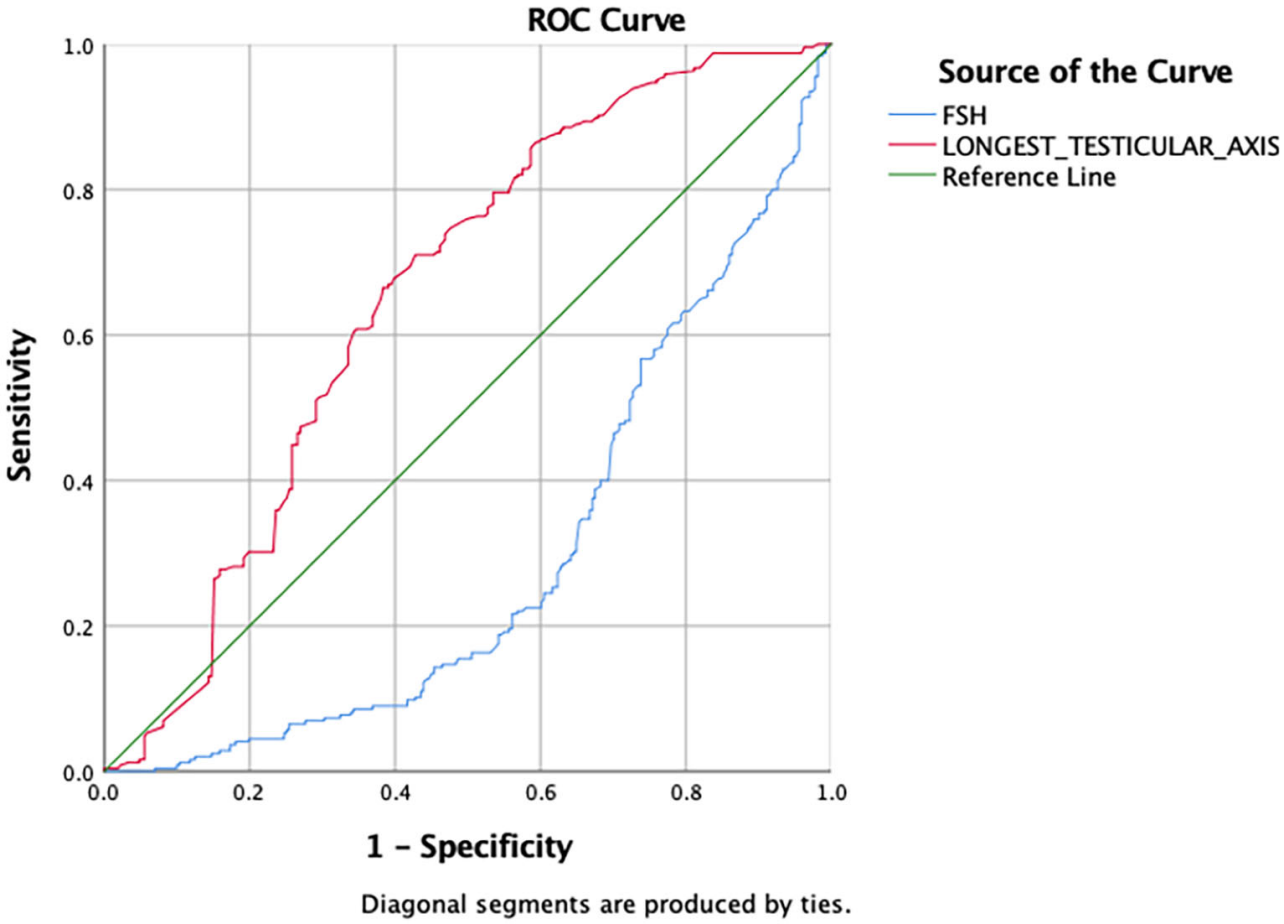
Receiver operating characteristic (ROC) curve for serum hormone level and longest testicular axis to predict sperm retrieval success. FSH, follicle stimulating hormone.

Receiver operating characteristic (ROC) curve analysis was performed. The cut- off for FSH 7.76 mIU/mL (sensitivity: 60.1%, specificity: 63.3%), p<0.001); longest testicular axis length 3.89 cm (sensitivity: 33.6%, specificity: 41.6%, p<0.001) ( Figure 1). Area under the curve for FSH and longest testicular axis was 0.293 (0.248-0.337, p<0.001) and 0.655 (0.607-0.702, p<0.001), respectively ( Figure 2).

### Subgroup analysis to determine OA vs. NOA

A subgroup analysis was performed to determine the clinical parameters that predicted the diagnosis of OA or NOA in men with azoospermia. The results are presented in Table 3. Higher serum levels of FSH and LH, varicocele, and a shorter testicular axis were significantly associated with the diagnosis of NOA. There were no significant differences in age, marriage duration, and previous illnesses, such as diabetes and UDT, regarding the diagnosis of OA or NOA. Logistic regression analysis revealed that FSH, varicocele, and longest testicular axis were independent risk factors for NOA ( Table 4). This logistic regression model comprised 42.0% of all possible factors for determining OA or NOA diagnosis, as indicated by an R ^2^ value of 0.420.

**Table 3.** Bivariate analysis of potential diagnostic factors for obstructive azoospermia *vs.* non-obstructive azoospermia.

Diagnostic factors		Obstructive azoospermia (n=163)	Non-obstructive azoospermia (n=354)	p-value
**Age (years)**		37 (22–67)	36 (25–65)	0.153 ^a^
**Marriage duration (years)**		5 (1–17)	5 (1–25)	0.851 ^a^
**Diabetes**	**Yes**	9 (47.36)	10 (52.64)	0.138 ^c^
	**No**	154 (30.92)	344 (69.08)	
**UDT**	**Yes**	4 (28.57)	10 (71.43)	0.534 ^b^
	**No**	159 (31.61)	344 (68.39)	
**Varicocele**	**Yes**	35 (12.50)	245 (87.50)	<0.001 ^c^
	**No**	128 (54.01)	109 (45.99)	
**FSH (mg/dL)**		5.89 (0.66–51.7)	11.68 (0.11–0.94.40)	<0.001 ^a^
**LH (mg/dL)**		4.07 (0.10–20.08)	5.07 (0.07–43.85)	<0.001 ^a^
**Testosterone (mmol/dL)**		3.96 (0.02–6.80)	3.56 (0.03–6.90)	0.201 ^a^
**Longest testicular axis (cm)**		4.30 (2.00–9.70)	3.49 (1.21–8.80)	<0.001 ^a^

Abbreviations: UDT, undescended testes; FSH, follicle-stimulating hormone; LH, luteinizing hormone.

^a^ Mann–Whitney test.

^b^ Fisher exact test.

^c^ Chi-square test.

**Table 4.** Linear regression analysis for potential clinical parameters to predict non-obstructive azoospermia diagnosis.

Clinical parameters	Coefficient	OR	95% CI	p Value
**FSH**	0.071	1.074	1.038-1.111	<0.001
**LH**	0.017	1.017	0.936-1.105	0.688
**Longest testicular axis**	-0.381	0.683	0.593-0.786	<0.001
**Varicocele**	2.022	7.553	4.695-12.151	<0.001

Abbreviations: FSH, follicle-stimulation hormone; LH, luteinizing hormone; OR, odds ratio; CI, confidence interval.

R ^2^ = 0.420.

**Figure 3.**
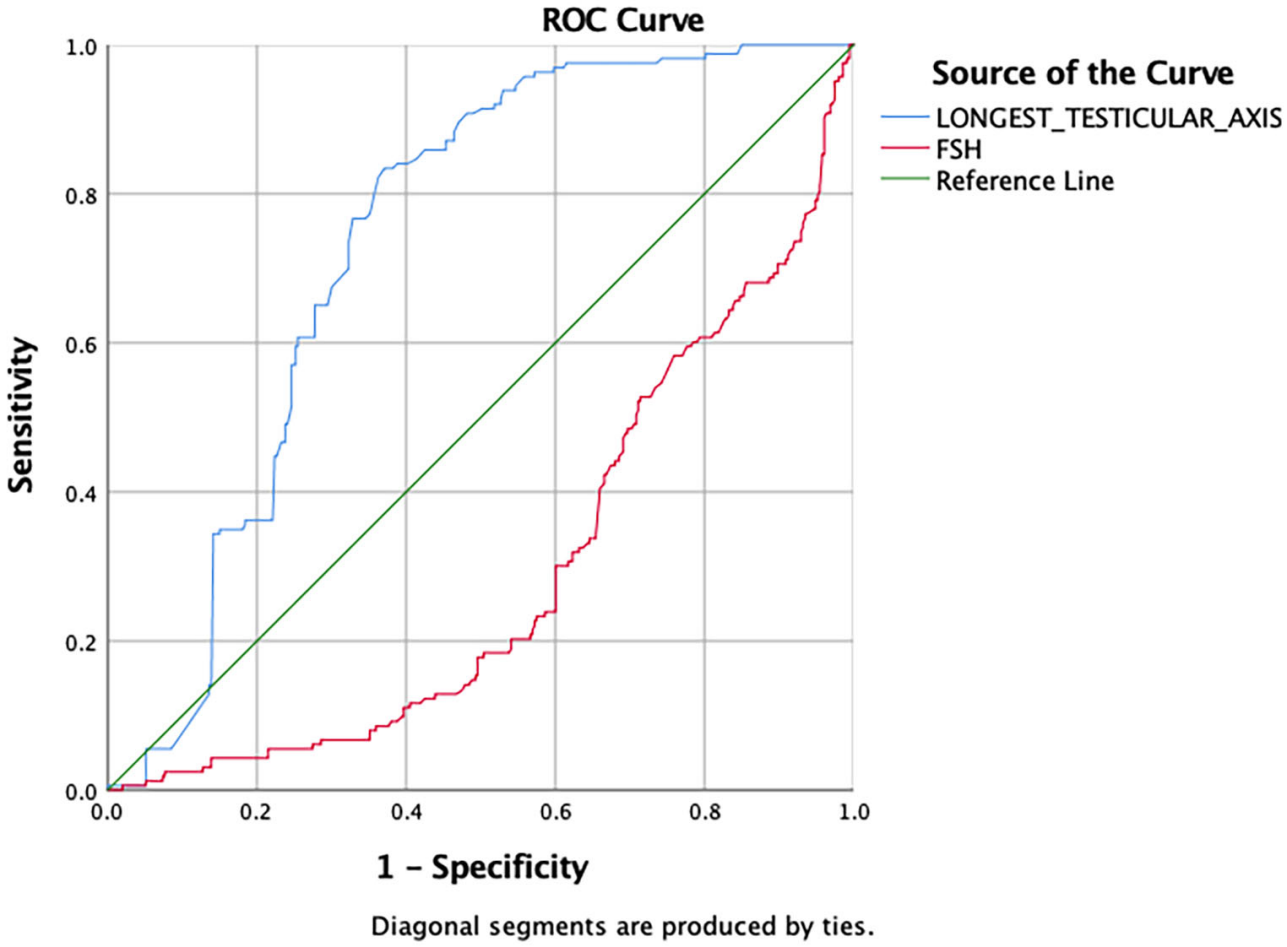
Receiver operating characteristic (ROC) curve for serum hormone levels and the longest testicular axis to predict the differentiation between OA and NOA. FSH, follicle stimulating hormone; OA, azoospermia; NOA, non-obstructive azoospermia.

ROC curve analysis was performed. For the diagnosis of NOA, the FSH cut-off value was 7.45 mIU/mL (sensitivity: 31.3%, specificity: 37.7%, p<0.001); longest testicular axis length 3.85 cm (sensitivity: 76.7%; specificity: 65.4%, p<0.001). Area under the curve for FSH and longest testicular axis were 0.707 (0.660-0.753, p<0.001) and 0.265 (0.222-0.307, p<0.001), respectively ( Figure 3).

### Subgroup analysis of clinical parameters to predict sperm retrieval success in NOA

A subgroup analysis was performed to determine the clinical parameters for predicting sperm retrieval success in patients with NOA. A total of 354 patients with NOA were included in the analysis. The results are presented in Table 5. Among men with NOA, a varicocele is a significant determinant of sperm retrieval success.

**Table 5.** Bivariate analysis of sperm retrieval success in non-obstructive azoospermia.

Clinical parameters		Successful Sperm Retrieval N=83	Unsuccessful Sperm Retrieval N=271	p-value
**Age (years)**		36 (25–54)	36 (25–65)	0.461 ^a^
**Marriage duration (years)**		5 (1–20)	5 (1–25)	0.647 ^a^
**Diabetes**	**Yes**	2 (20.00)	8 (80.00)	0.555 ^c^
	**No**	81 (23.54)	263 (76.46)	
**UDT**	**Yes**	3 (30.00)	7 (70.00)	0.445 ^b^
	**No**	80 (23.25)	264 (76.75)	
**Varicocele**	**Yes**	42 (17.14)	203 (82.96)	<0.001 ^c^
	**No**	41 (37.61)	68 (62.39)	
**FSH (mg/dL)**		10.48 (1.99–94.40)	11.9 (0.11–69.10)	0.309 ^a^
**LH (mg/dL)**		5.13 (0.11–43.85)	5.03 (0.07–43.15)	0.898 ^a^
**Testosterone (mmol/dL)**		3.69 (0.04–8.20)	3.45 (0.03–7.90)	0.718 ^a^
**Longest testicular axis (cm)**		3.57 (1.50–8.80)	3.47 (1.21–8.61)	0.697 ^a^

Abbreviations: UDT, undescended testicle; FSH, follicle-stimulating hormone; LH, luteinizing hormone.

^a^ Mann–Whitney test.

^b^ Fisher exact test.

^c^ Chi-square test.

Table 6 displays a summary of FSH and longest testicular axis cut-offs for all subgroups. The cut-off value for FSH and longest testicular axis to determine overall sperm retrieval success and predicting NOA diagnosis is not very different. FSH has better sensitivity and specificity in predicting overall sperm retrieval success compared to longest testicular axis, on the other hand, the longest testicular axis has better sensitivity and specificity in predicting NOA diagnosis.

**Table 6.** Summary of FSH and longest testicular axis cut-offs for all subgroups.

Subgroups	FSH	Sensitivity	Specificity	Longest testicular axis	Sensitivity	Specificity
**Overall sperm retrieval success**	7.76	60.1	63.3	3.89	33.6	41.6
**OA vs. NOA**	7.45	31.3	37.7	3.845	76.7	65.4

FSH, follicle-stimulating hormone; OA, obstructive azoospermia; NOA, non-obstructive azoospermia.

## Discussion

The management of male infertility is challenging because of multifactorial causes. The leading cause of male infertility is idiopathic, comprising 30–40% of all azoospermia. ^1^ Azoospermia is one of the most challenging andrological conditions, and is the most severe form of male infertility. According to the European Association of Urology guidelines, only 10% of all infertile male patients present with azoospermia. ^1^ However, in Indonesia, 53% of infertile patients presented with azoospermia, 46% had at least one normal semen parameter, and 4.9% had normal semen parameters. ^12^

Up to 15% of infertile men have azoospermia, which affects approximately 1% of all men. Azoospermia is roughly divided into NOA and OA, depending on the capacity of the testes to generate and distribute spermatozoa. ^13^ The diagnosis of OA varies among studies. From clinical parameters (normal testicular volume and FSH level), ^14^ performing PESA or testicular biopsy may be indicated. ^14^^–^^16^ In this study the diagnosis of OA is established by successful PESA procedure.

Numerous factors of testicular or pre-testicular origin may cause NOA. Genetic conditions such as Klinefelter syndrome and Y chromosome microdeletions, congenital conditions such as cryptorchidism, exposure to radiotherapy and chemotherapy, genital trauma, and complex infections such as mumps orchitis are among the testicular causes of NOA. The primary endocrine factors that lead to pretesticular hypogonadism are abnormalities of the hypothalamic-pituitary-gonadal axis. Additionally, up to 15% of NOA cases may be idiopathic. ^17^

Multivariate analysis revealed that FSH, varicocele, and longest testicular axis were the three independent variables predicting sperm retrieval success and NOA diagnosis. Varicocele was the only independent variable that determined sperm retrieval success in patients with NOA. Another study by Yang *et al.*, also reported that FSH is an independent risk factor in predicting the sperm retrieval rate (SRR) in NOA with a sensitivity of 0.70 (0.66-0.73) and specificity of 0.62 (0.58-0.66). ^18^ The result of a study by Salehi *et al.*, are in line with those of the present study, stating that high levels of FSH and small testicular volume were associated with a lower chance of successful sperm retrieval. ^19^ A systematic review by Major *et al.*, also showed that FSH levels exhibit an inverse relationship with the SRR in conventional TESE. Another study confirmed that varicocele repair is beneficial for increasing the SRR and improving the testicular histopathological pattern (p<0.001) regardless of the patient’s FSH level. ^20^

While the surgeon’s experience and laboratory expertise in dissecting and processing the testicular parenchyma can undoubtedly influence the success of sperm retrieval, many other predictors have also been investigated, such as clinical profiles, Klinefelter syndrome status, cryptorchidism, paternal age, testicular volume, and laboratory panels (FSH, inhibin B, Y-chromosome microdeletion, and surgery). ^21^

To our knowledge, this is the first study to develop a predictive model for successful sperm retrieval in Indonesia. A limitation of this study is that the R2 value of multivariate of the linear regression for sperm retrieval success and NOA diagnosis were only 0.410 and 0.420, respectively, indicating that the factors investigated in this study only comprise 41.0% and 42.0% of all possible factors to predict sperm retrieval success. The authors recommend that further high-quality studies be undertaken to assess other factors that predict sperm retrieval success.

## Conclusions

FSH, LH, longest testicular axis, and varicocele are among the clinical parameters that are useful for predicting overall sperm retrieval success and NOA diagnosis. However, varicocele is the only clinical parameter that significantly predicts sperm retrieval success in patients with NOA. High-quality studies are required to assess the other predictors of sperm retrieval success.

## Data Availability

Open Science Framework: Clinical Parameters as Predictors For Sperm Retrieval Success In Azoospermia: Experience From Indonesia. https://doi.org/10.17605/OSF.IO/2EAU4. ^22^ Data are available under the terms of the Creative Commons Zero “No rights reserved” data waiver (CC0 1.0 Public domain dedication).
