# Supplementary material for: Clinical parameters as predictors for sperm retrieval success in azoospermia: experience from Indonesia
Source: F1000Res. 2023 Dec 6;12:1567. [Version 1] doi: 10.12688/f1000research.141969.1 (PMC10905114; doi:10.12688/f1000research.141969.1)
Supplement: Peer review history

# Reviewer report: Reviewer response for version 1

**DOI:** 10.5256/f1000research.155459.r235153

**Published:** 2024-02-07

**Authors:** Dragos Puia

**Affiliations:** 1 University for Medicine and Pharmacy, Iasi, Romania

After reading this interesting manuscript, I have some questions and suggestions.

 1. Paragraph 2 in the Methods subchapter represents Results

 2. Why patients with severe systemic diseases (ASA III) underwent an elective surgery?

 3. How many sperm analysis had the patients performed before receiving the diagnosis of azoospermia (current guideline recommends at least 2 tests)

 4. In Table 1, please give SD and I would suggest replacing "marriage duration: with "infertility duration"

 5. How was obstructive azoospermia differentiated from non-obstructive? Please give details

 6. I think this study has severe limitations resulting from the fact that many important predictive factors like genetic test or Inhibin B levels have not been evaluated.

Is the work clearly and accurately presented and does it cite the current literature?

Yes

If applicable, is the statistical analysis and its interpretation appropriate?

Yes

Are all the source data underlying the results available to ensure full reproducibility?

Yes

Is the study design appropriate and is the work technically sound?

Yes

Are the conclusions drawn adequately supported by the results?

Yes

Are sufficient details of methods and analysis provided to allow replication by others?

Yes

Reviewer Expertise:

Urology, male infertility

I confirm that I have read this submission and believe that I have an appropriate level of expertise to confirm that it is of an acceptable scientific standard, however I have significant reservations, as outlined above.

# Reviewer report: Reviewer response for version 1

**DOI:** 10.5256/f1000research.155459.r229809

**Published:** 2024-02-06

**Authors:** Doddy M. Soebadi

**Affiliations:** 1 Department of Urology, Faculty of Medicine, Universitas Airlangga, Surabaya, East Java, Indonesia

This is a simple but important study, that gives us, especially in this region, that we have data how many percent we could predict the result of this procedure for azoospermic couple.

 A very simple (only physical examination and some laboratory examination) and practical, that every physician treating aziospermic patient has evidence to inform the couple the prediction of the procedure.

 More details and may be if possible a random and more scientific research are needed to support this findings.

Is the work clearly and accurately presented and does it cite the current literature?

Yes

If applicable, is the statistical analysis and its interpretation appropriate?

I cannot comment. A qualified statistician is required.

Are all the source data underlying the results available to ensure full reproducibility?

Yes

Is the study design appropriate and is the work technically sound?

Yes

Are the conclusions drawn adequately supported by the results?

Yes

Are sufficient details of methods and analysis provided to allow replication by others?

Yes

Reviewer Expertise:

Reproductive Urology.

I confirm that I have read this submission and believe that I have an appropriate level of expertise to confirm that it is of an acceptable scientific standard.
